# Hypertonic saline in people with cystic fibrosis: review of comparative studies and clinical practice

**DOI:** 10.1186/s13052-021-01117-1

**Published:** 2021-08-06

**Authors:** Vito Terlizzi, Eleonora Masi, Michela Francalanci, Giovanni Taccetti, Diletta Innocenti

**Affiliations:** 1 Department of Paediatric Medicine, Cystic Fibrosis Regional Reference Center, Anna Meyer Children’s Hospital, Viale Gaetano Pieraccini 24, 50139 Florence, Italy; 2 Rehabilitation Unit, Anna Meyer Children’s Hospital, Florence, Italy

**Keywords:** Mucolytic agents, Dornase alfa, Mannitol, Physiotherapy

## Abstract

Cystic fibrosis (CF) is a multisystem disorder, caused by mutations in the CF transmembrane conductance regulator (CFTR) gene. These cause a reduced secretion of chloride, a marked absorption of sodium and, therefore, of water, through the epithelium, resulting in the formation of thickened secretions in organs such as lung or pancreas. These viscous secretions lead to airway obstruction, chronic infection and inflammation resulting in progressive lung damage, bronchiectasis and eventual respiratory failure. Although the average life expectancy has increased over the last 30 years, lung disease is the most common cause of death in people with CF. For these reasons, the improvement of sputum clearance is a major therapeutic aim in CF and early initiation of airway clearance is widely recommended and implemented. Symptomatic mucolytic therapy today is mainly based on inhalation of DNase, hypertonic saline or mannitol, in combination with physiotherapy. Mucolytic agents break down the gel structure of mucus and therefore decrease its elasticity and viscosity, reducing the pulmonary exacerbation frequency and to improve and stabilize lung function. Nevertheless, high quality studies comparing these mucolytic drugs are still few, and the individual experiences of patients and caregivers explain the high variability of their use globally. This review will summarize the current knowledge on hypertonic saline in the treatment of CF lung disease. Furthermore, we report the real-world prescription of inhaled mucolytic agents in CF.

## Introduction

Cystic Fibrosis (CF) is a genetic disease caused by mutations in the CF transmembrane conductance regulator (CFTR) gene encoding the CFTR protein. This protein is an ion channel that carries chloride ions and water across cell membranes. It is also involved in regulating the functioning of other important channels in mucociliary clearance and innate defense mechanisms [1]. To date, therapeutic advances have resulted in a notable increase in life expectancy [2]. Recommended beneficial treatments include pancreatic enzymes, airway clearance, mucolytics, inhaled antibiotics, anti-inflammatory agents and CFTR modulators [3, 4]. CF-causing mutations are classified into 6 categories, according to their impact on the production, trafficking, functioning or stability of the CFTR channel [5–8]. Mutations belonging to classes I, II and III usually result in little to no CFTR activity, leading to severe clinical outcomes, whilst mutations from classes IV, V and VI allow significant residual CFTR function leading to milder phenotypes [5–8]. These alterations affect the glands that produce mucus, sweat, saliva, tears, and digestive enzymes. In normal conditions, mucus acts as a barrier to protect the airways by trapping inhaled particles and pathogens, thereby preventing infections. Mucus is a complex and viscous secretion containing proteins, lipids, ions and water. In patients with CF, the defect of the CFTR protein causes a reduced secretion of chloride and a marked absorption of sodium, and therefore of water, through the epithelium, resulting in the formation of thickened secretions in organs such as the pancreas or lung. These viscous secretions lead to airway obstruction, chronic infection and inflammation resulting in progressive lung damage, bronchiectasis and eventual respiratory failure [9, 10]. In healthy subjects, the main component of mucus is a glycoprotein called mucin, but the secretions of people with CF contain very little mucin. In fact, pus, polymerized DNA and filamentous actin (F-actin) proteins derived from dead inflammatory cells and epithelial cells trapped in mucus prevail [11]. This has important therapeutic implications, as substances that act against mucin components are ineffective.

Nowadays lung disease remains the most common cause of death in people with CF. [1, 2] For this reason, acting against the accumulation of secretions and the evolution of lung damage is one of the best therapeutic strategies. Mucolytic agents are drugs that reduce mucus viscosity by degrading mucin polymers, DNA or F-actin in the airways secretions. This allows for better elimination of sputum with coughing [12].

Symptomatic mucolytic therapy today is mainly based on inhalation of DNase, hypertonic saline or mannitol in combination with physiotherapy.

Mucolytic agents break down the gelatinous structure of mucus and therefore decrease its elasticity and viscosity, reducing the pulmonary exacerbation frequency and to improve and stabilize lung function. However, high quality studies comparing these mucolytic drugs are still lacking, and the individual experiences of patients and caregivers explain the high variability of their use globally.

This review summarizes the current knowledge on hypertonic saline in the treatment of CF lung disease. Furthermore, we report the real-world prescription of inhaled mucolytic agents in CF. A systematic review of peer-reviewed literature was conducted using Medline/PubMed, Cochrane and Google Scholar.

## Saline hypertonic

### Generality

The use of hypertonic saline solution represents a therapeutic adjuvant weapon to respiratory physiotherapy, both in the long term and in the course of respiratory exacerbation [13]. Furthermore, it is a low-cost and easily reproducible formulation. Already in 1995 Dasgupta et al. showed that in vitro the addition of 3% hypertonic saline on the mucus surface improved its clearance, with an even greater effect than Dornase alfa [14]. There are several mechanisms underlying this effect:

- Breaking of ionic bonds with reduction of “cross-linking” and mucus viscosity [15];
- Increased ionic concentration and conformational change of mucus resulting in more effective mucociliary clearance [16];
- Increased osmotic flow of water in the mucus layer, with rehydration of secretions [16].
- In the long term, the improvement in mucociliary function reduces the bacterial load and the degree of chronic inflammation within the airways [13].

### Comparative studies between hypertonic saline solution and placebo or other drugs

Three trials examined the effect of using hypertonic saline, from 3 to 7% and used for 4 weeks, on FEV_1_ values compared to a group of patients who took isotonic saline (NaCl 0.9%). In a study of Eng et al., 52 CF patients with a mean age of 16.2 years and with moderate-to-severe lung disease took 6% hypertonic solution for 2 weeks. At the end of this period, a significant increase in FEV_1_ was observed compared to the control group (15.0 ± 16.0% vs 2.8 ± 13%, p 0.004). In addition, patients reported a subjective improvement in the beneficial effects of respiratory physiotherapy and greater tolerance to physical exercise [17]. On the contrary, Amin et al. did not show significant improvements in spirometric parameters or in the quality of life in 20 paediatric patients with normal respiratory function, treated for 4 weeks with hypertonic solution. The improvement parameter in the treated subjects was represented by the lung clearance index (LCI) (difference of 1.16 points, p 0.016) [18]. It is a lung function outcome that has been shown to be more sensitive than spirometry, to correlate with airway changes seen on high-resolution computed tomography and to detect significant treatment effects in randomized controlled trials or in preschool-aged CF patients [19–21].

Elkins et al. in 2006 evaluated the effects of hypertonic saline at 7% in 164 patients aged > 6 years treated for 48 weeks, in a double-blind, randomized controlled trial. There were no differences in the spirometric parameters expressed in % (FEV_1_, FVC, FEF _25–75_) in the two groups, while the treated group had an increase in absolute values ​​from 4 to 48 weeks of treatment (68 ml for FEV1, 82 ml for FVC). Furthermore, in the treated group there was a lower frequency of respiratory exacerbations (mean number 0.39 vs 0.89, p 0.02), shorter duration of the same (6 days vs 17 days), fewer days of absence from school or from the workplace. Patients also taking Dornase alfa showed no difference in parameters compared to patients taking hypertonic solution alone. The therapy was well tolerated, coughing was frequent shortly after the introduction of the drug but tended to disappear over time. However, premedication with salbutamol was recommended [22].

The combined analysis of the 3 above studies showed, in patients aged > 12 years, an increase in FEV_1_ values after 4 weeks of treatment (MD 3.44, 95% Cl 0.67–6.21) with a low degree of evidence [13].

Recently, Ratjen at al assessed the effect of inhaled hypertonic saline on LCI _2.5_, in CF children aged 3–6 years. This was a randomised, double-blind, placebo-controlled trial, including 150 children, treated for 48 weeks with inhaled 7% hypertonic saline or 0.9% isotonic saline nebulised twice daily.

Inhaled hypertonic saline improved the LCI _2.5_ and could be a suitable early intervention in CF. [23]

In the same way, Stahl et al. demonstrated that preventive inhalation of 6% hypertonic saline in infants < 4 months of age results in a significant improvement in LCI compared to subjects treated with isotonic solution after 52 weeks of therapy (− 0.6 vs − 0.1, *p* < 0.05). In addition, there was also an improvement in weight (p < 0.05) while there were no differences regarding the number of respiratory exacerbations or the MRI scores of the chest. The therapy was well tolerated [24].

Other studies have evaluated the improvement of mucociliary clearance as an outcome, demonstrating greater benefits from the use of hypertonic saline than isotonic solution, also in children with mild CF lung disease [16, 25, 26] Furthermore, a more recent study demonstrated the ability of the 7% hypertonic solution to facilitate expectoration in paediatric age, especially in children aged < 11 years, facilitating the identification of pathogenic bacteria [27]. On the other hand, no significant improvements were shown from the use of a more concentrated hypertonic solution, for example at 12% [16]. Similarly, three trials evaluated the improvement of the quality of life of both patients and parents as an outcome [18, 22, 28]. The combined analysis did not demonstrate a greater effect of the hypertonic solution compared to the group treated with isotonic solution (MD 1.62, 95% Cl − 169-4.92).

The efficacy of hypertonic saline during respiratory exacerbations was evaluated in a randomized and controlled trial on 132 adults with CF [29]. The addition of 7% hypertonic saline did not reduce the duration of hospitalization or distance the occurrence of the subsequent exacerbation but helped restore FEV_1_to pre-hospitalization values and positively influenced the reduction of symptoms such as dyspnoea, congestion and normalization of sleep. Regarding the efficacy of 7% saline hypertonicity in reducing the rate of respiratory exacerbations in children aged < 6 years, Rosenfled et al. carried out a randomized controlled and multicentre trial on 158 CF patients. At the end of 48 weeks of treatment, the treated group showed no benefit from the therapy performed compared to the control group. Similarly, there were no differences in secondary outcomes (auxological parameters, respiratory symptoms or oxygen saturation). No major adverse events were identified in the treated group [28].

### Hypertonic saline solution with hyaluronic acid

The addition of 0.1% hyaluronic acid to the hypertonic solution was considered to reduce adverse events such as cough or the sensation of salty taste reported by patients. In 2010 Buonpensiero et al. evaluated the effect of adding hyaluronic acid to 7% hypertonic saline in 20 CF children (mean age: 13 years). The same patients took 7% hypertonic saline solution on non-consecutive days in order to assess the differences in tolerance and palatability. The addition of hyaluronic acid had an improving effect on the outcomes considered. No differences in respiratory function tests were identified [30]. In 2016 Brivio et al. evaluated the effect of adding hyaluronic acid to the 7% hypertonic solution in reducing inflammation of the airways in CF children, measured as the level of cytokines in the sputum. No significant differences were identified in the two groups of patients nor differences in the prevalence of symptoms such as cough, palatability and pharyngeal irritation in lung tests performed after 28 days [31].

### Adverse events

Six trials reported the appearance of adverse events after the use of hypertonic solution: it should be considered, however, that none of the results were statistically significant compared to the patients who took isotonic solution and in conclusion the quality of evidence was very low. However, a drop in FEV_1_ is reported in the first minutes after the administration of hypertonic solution or adverse events such as cough and increased sputum production [13].

## Discussion

The systematic reviews conclude that there is no superiority of hypertonic saline than other mucolytic agents [13, 32, 33]. Guidelines generally do not provide recommendations on which to start first. Since these agents have different mechanisms of action, it is possible benefit from the use of more than one at the same time [34]. We think that the choice of the inhalation mucolytic therefore requires careful clinical evaluation and sharing with patients and their families, having considered all available options. Hypertonic saline solution can be useful in paediatric age to promote the expectoration or, in most cases, especially with the progression of the disease, combined with other mucolytic agents, such as dornase alfa or inhalation preparations such as antibiotics, bronchodilators and corticosteroids. Specific mucolytic agents for CF patients and related devices represent a limited number. The main used in CF are reported in Table 1.

**Table 1:** Main mucolytic agents used in Cystic Fibrosis, with indication of the methods of preparation and storage, devices recommended and indications for use

Type of mucolytic	Indication for age (years)	Formulation, preparation and dosage	Conservation	Inhalation device
Hypertonic saline solution 7% of NaCl + hyaluronic acid	> 6	Ready-made 5 ml vial 2vv/die	Room temperature	- Jet nebulizer - Mesh nebulizer
Hypertonic saline solution 7% of NaCl + sodium bicarbonate	> 6	Ready-made 5 ml vial 2vv/die	Temperature 5 °C - 25 °C	- Jet nebulizer - Mesh nebulizer
Hypertonic saline solution 7% of NaCl	> 6	Ready-made 4 ml vial 2vv/die	Temperature 4 °C–25 °C.	- Jet nebulizer - Mesh nebulizer
Hypertonic saline solution 6% of NaCl	> 6	Ready-made 4 ml vial 2vv/die	Room temperature	- Jet nebulizer - Mesh nebulizer - Ultrasonic nebulizers
Hypertonic saline solution 3% of NaCl	Every age	Ready-made 3–5 ml vial	Room temperature	- Jet nebulizer - Mesh nebulizer - Ultrasonic nebulizers
Mannitol 40 mg	> 18	10 capsules to be inhaled with specific device 400 mg × 2 times/die	Temperature < 30 °C In absence of humid environment	Specific inhaler
Dornase alfa	> 5	Ready-made 2.5 ml vial 1 time/die > 21 years 2 times/die (for severe patients)	Temperature: 2 °C - 8 °C max 30 °C for 24 h	- Jet nebulizer - Mesh nebulizer - Adaptive aerosol delivery system. No ultrasonic nebulizers

Few studies compared the effectiveness of the hypertonic solution to dornase alfa; Suri et al. carried out a cross-over trial on 48 children, comparing the effect on FEV_1_ of DNAse and hypertonic saline at 7% (5 ml twice a day), used for 12 weeks. DNAse determined a much greater increase in FEV_1_ (16% vs 3%) albeit at higher economic costs. There was no significant difference between the two groups about the number of respiratory exacerbations [35]. A subsequent study by Ballmann et al. was conducted on 14 children with mild or moderate lung disease who took DNAse or hypertonic saline for 3 weeks with a subsequent 3-week washout period. Both drugs determined an increase of the FEV_1_: 9.3% in the DNAse group vs 7.7 in the 7% hypertonic solution group. Patients treated with DNAse more likely showed a clinically relevant increase in FEV_1_ (> 10%) but without statistically significant differences compared to the group treated with hypertonic solution (OR 1.00) [36]. Similarly, Adde et al. showed no changes in FEV_1_, bacterial colonization and symptoms in two groups of children (# 18) treated for 2 weeks with DNAse and 6% hypertonic saline. Both drugs were well tolerated [37]. The combined analysis shows no differences between treatments after 3 weeks of therapy (very low level of evidence), but a greater effect of DNAse after 3 months of therapy [13, 32]. Saline hypertonic is certainly a cheaper drug but requires longer administration time than DNAse and this can affect the compliance of treatment [35, 36] There are no differences in the rate of adverse events, although acute bronchospasm remains a possible finding after administration of hypertonic saline.

In children under 5 years of age, dornase alfa and the solution hypertonic saline should be considered based on the assessment individual clinic [38].

The UK guidelines (NICE, 2018) recommend the dornase alfa as the first choice in routine treatment. If the clinical response is inadequate, hypertonic saline is also proposed alone or in combination with dornase alfa.

The guidelines published by the CF Foundation recommend long-term use term of dornase alfa to preserve lung function and reduce exacerbations in patients with lung disease of moderate to severe degree. Dornase alfa is also recommended for patients with mild or asymptomatic lung disease or in children under the age of 5 years, based on individual assessment [39, 40]. Use chronic hypertonic saline is recommended from 6 years of age [39].

On the other hand, only one trial has been published comparing the effect of 6% hypertonic solution and mannitol in 12 CF patients. The main outcome evaluated was the ability to improve mucus clearance, which was found to be insufficient for both drugs. A fall in FEV_1_ was reported for both, mostly after the use of mannitol but without statistically significant differences (7.3% ± 2.5% vs 5.8% ± 1.2%) [41].

Guidelines of European CF Society, consider dornase alfa as the mucolytic agent to be used in long-term maintenance therapy, indicate the potential use of hypertonic saline in patients with moderate to severe pulmonary impairment and the use of mannitol to improve lung function and to reduce the treatment times [38]. Inhalation of mannitol is also recommended in adults with rapidly declining lung function or in case of lack response to other medications [41].

To date there is not a clear indication in asymptomatic CF patients or with mild disease [26]. In these cases, it is important to involve the patient and her family in common decision-making process and identify the best device, in order to integrate it in the real program. This choice depends on the age of the patient, the severity of the lung disease, the amount and quality of inhaled drugs in the treatment plan.

The role of the care team is to select together with the patient the best drug for the clinical characteristics of the patient, and the devices with which to administer it. Since saline hypertonicity and mannitol can produce bronchospasm during or after inhalation, a premedication with bronchodilator drugs and the execution of the tolerance test are recommended [39]. About the chronology with which to inhale the mucolytic agents compared to performing respiratory physiotherapeutic release there is not yet sufficient scientific evidence.

A recent review on hypertonic saline solution suggests that the timing of the inhalation does little or no difference in lung function [42]. However, inhalation before or during airway removal techniques can maximize effectiveness and perceived satisfaction by the patient. The long-term effectiveness of the saline solution hypertonic was established with bi-daily administrations; however, if only one dose per day is tolerated, that is the time in which to perform the inhalation is indicated on the tolerance and the patient preference [42].

To date, the best timing for inhalation of dornase alfa is still debated. It seems that the drug requires at least 30 min of time inside the lung to show changes in sputum viscosity. It follows therefore that better results would be expected if at least thirty minutes were expected before performing the drainage session secretions. It has been demonstrated on a sample of young subjects that there was no difference when dornase alfa was inhaled shortly before night rest or later in the day, earlier of the execution of respiratory physiotherapy [43].

It can be suggested not to inhale immediately before unblocking respiratory physiotherapy, also leaving to the patient the choice of a time that best suits the life style and subjective efficacy in relation to the drug [44].

We propose a chronological order of execution of the various therapies in Table 2. Nevertheless, the listed therapies are variously associated in clinical practice and there is no agreement on the correct sequence. For example, inhalation antibiotic therapy can also be performed at the end of the physiotherapy cycle [39]. Finally, we recommend an annual verification of the prescription, in order to optimize the therapy, increase compliance and solve any problems in progress (Table 3).

**Table 2:** Practical example of the temporal sequence for the execution of daily inhalation therapy

1. Bronchodilator;	
2. Wait 5–15 min;	
3. Mucolytic such as saline hypertonic or mannitol;	
4. Airway clearance techniques;	
5. Inhaled antibiotics;	
6. Long-acting bronchodilators / inhaled steroids;	
7. Dornase alfa.

**Table 3:** Annual check list

Annual check-list						
Patient’s name			YEAR			
ITEMS	Date	yes/no	Date	yes/no	date	yes/no
Have the objectives of aerosol therapy been clarified?						
Has the action of the individual drugs been explained?						
Does the person agree on the prescribed therapy?						
Have the drugs in the prescription been checked?						
Have the type of dilutions been checked?						
Has the patient’s nebulizer equipment been checked?						
It has been asked if they are used?						
Have they been checked by the physiotherapist?						
Were verbal instructions given?						
Has the patient been given the opportunity to directly show how to manage and use the devices?						
Written instructions: have they been delivered?						
Written instructions: have they been understood?						
Has the patient been given time and means to reformulate the educational and technical aspects, express doubts, ask for clarification?						
Was the opinion on the feeling of effectiveness of the drug asked?						
Has the duration of aerosol therapy with individual drugs been asked?						
Is the aerosol completely taken?						
Has the logic of the drug intake sequence in relation to physiotherapy been clarified?						
Has the last replacement of the hose, filters, head, nebulizer, engineering review, etc. been checked?						
Is the difference between cleaning and disinfection clear?						
Has it been investigated how cleaning and disinfection are done?						
And the frequency of cleaning and disinfection?						
Operator signature						

Taken and translated from: Gruppo di Studio Multidisciplinare della SIFC sull’Aderenza Terapeutica in Fibrosi Cistica. “La valutazione e l’implementazione dell’aderenza allaterapia inalatoria e alla fisioterapia respiratoria nella Fibrosi Cistica”. Orizzonti -Supplemento al n.2–2016: 19

## Conclusions

Mucolytic agents are part of a comprehensive treatment strategy contributing to improvement in lung function and quality of life of CF patients.

To date there is no superiority of hypertonic saline than other mucolytic agents. Hypertonic saline preceded by a bronchodilator is an inexpensive, safe, and effective additional therapy for patients with CF.

## Data Availability

All reported data are available.

## References

[1] Bell SC, Mall MA, Gutierrez H, Macek M, Madge S, Davies JC, Burgel PR, Tullis E, Castaños C, Castellani C, Byrnes CA, Cathcart F, Chotirmall SH, Cosgriff R, Eichler I, Fajac I, Goss CH, Drevinek P, Farrell PM, Gravelle AM, Havermans T, Mayer-Hamblett N, Kashirskaya N, Kerem E, Mathew JL, EF MK, Naehrlich L, Nasr SZ, Oates GR, O'Neill C, Pypops U, Raraigh KS, Rowe SM, Southern KW, Sivam S, Stephenson AL, Zampoli M, Ratjen F (2020). The future of cystic fibrosis care: a global perspective. Lancet Respir Med.

[2] Kapnadak SG, Dimango E, Hadjiliadis D, Hempstead SE, Tallarico E, Pilewski JM, Faro A, Albright J, Benden C, Blair S, Dellon EP, Gochenour D, Michelson P, Moshiree B, Neuringer I, Riedy C, Schindler T, Singer LG, Young D, Vignola L, Zukosky J, Simon RH (2020). Cystic Fibrosis Foundation consensus guidelines for the care of individuals with advanced cystic fibrosis lung disease. J Cyst Fibros.

[3] Salvatore D, Terlizzi V, Francalanci M, Taccetti G, Messore B, Biglia C, Pisi G, Calderazzo MA, Caloiero M, Pizzamiglio G, Majo F, Cresta F, Leonetti G, De Venuto D (2020). Ivacaftor improves lung disease in patients with advanced CF carrying CFTR mutations that confer residual function. Respir Med.

[4] Middleton PG, Mall MA, Dřevínek P, Lands LC, McKone EF, Polineni D, Ramsey BW, Taylor-Cousar JL, Tullis E, Vermeulen F, Marigowda G, McKee CM, Moskowitz SM, Nair N, Savage J, Simard C, Tian S, Waltz D, Xuan F, Rowe SM, Jain R (2019). VX17-445-102 study group. Elexacaftor-Tezacaftor-Ivacaftor for cystic fibrosis with a single Phe508del allele. N Engl J Med.

[5] O'Sullivan BP, Freedman SD (2009). Cystic fibrosis. Lancet..

[6] Sofia VM, Surace C, Terlizzi V, Da Sacco L, Alghisi F, Angiolillo A, Braggion C, Cirilli N, Colombo C, Di Lullo A, Padoan R, Quattrucci S, Raia V, Tuccio G, Zarrilli F, Tomaiuolo AC, Novelli A, Lucidi V, Lucarelli M, Castaldo G, Angioni A (2018). Trans-heterozygosity for mutations enhances the risk of recurrent/chronic pancreatitis in patients with cystic fibrosis. Mol Med.

[7] Terlizzi V, Di Lullo AM, Comegna M, Centrone C, Pelo E, Castaldo G, Raia V, Braggion C (2018). S737F is a new CFTR mutation typical of patients originally from the Tuscany region in Italy. Ital J Pediatr.

[8] Terlizzi V, Tosco A, Tomaiuolo R, Sepe A, Amato N, Casale A, Mercogliano C, De Gregorio F, Improta F, Elce A, Castaldo G, Raia V (2014). Prediction of acute pancreatitis risk based on PIP score in children with cystic fibrosis. J Cyst Fibros.

[9] Knowles MR, Olivier KN, Hohneker KW, Robinson J, Bennett WD, Boucher RC (1995). Pharmacologic treatment of abnormal ion transport in the airway epithelium in cystic fibrosis. Chest..

[10] Dinwiddie R (2000). Pathogenesis of lung disease in cystic fibrosis. Respiration..

[11] Rose MC, Voynow JA (2006). Respiratory tract mucin genes and mucin glycoproteins in health and disease. Physiol Rev.

[12] Henke MO, Ratjen F (2007). Mucolytics in cystic fibrosis. Paediatr Respir Rev.

[13] Wark P, McDonald VM (2018). Nebulised hypertonic saline for cystic fibrosis. Cochrane Database Syst Rev.

[14] Dasgupta B, Tomkiewicz RP, Boyd WA, Brown NE, King M (1995). Effects of combined treatment with rhDNase and airflow oscillations on spinnability of cystic fibrosis sputum in vitro. Pediatr Pulmonol.

[15] Ziment I (1978). Respiratory pharmacology and therapeutics.

[16] Robinson M, Hemming AL, Regnis JA, Wong AG, Bailey DL, Bautovich GJ, King M, Bye PT (1997). Effect of increasing doses of hypertonic saline on mucociliary clearance in patients with cystic fibrosis. Thorax..

[17] Eng PA, Morton J, Douglass JA, Riedler J, Wilson J, Robertson CF (1996). Short-term efficacy of ultrasonically nebulized hypertonic saline in cystic fibrosis. Pediatr Pulmonol.

[18] Amin R, Subbarao P, Jabar A, Balkovec S, Jensen R, Kerrigan S, Gustafsson P, Ratjen F (2010). Hypertonic saline improves the LCI in paediatric patients with CF with normal lung function. Thorax..

[19] Aurora P, Gustafsson P, Bush A, Lindblad A, Oliver C, Wallis CE, Stocks J (2004). Multiple breath inert gas washout as a measure of ventilation distribution in children with cystic fibrosis. Thorax..

[20] Shaw M, Khan U, Clancy JP, Donaldson SH, Sagel SD, Rowe SM, Ratjen F (2020). PROSPECT Investigators of the Cystic Fibrosis Foundation Therapeutics Development Network. Changes in LCI in F508del/F508del patients treated with lumacaftor/ivacaftor: Results from the prospect study. J Cyst Fibros.

[21] Terlizzi V, Amato F, Castellani C, Ferrari B, Galietta LJV, Castaldo G, et al. Ex vivo model predicted in vivo efficacy of CFTR modulator therapy in a child with rare genotype. Mol Genet Genomic Med. 2021:e1656. 10.1002/mgg3.1656 PMC8123755 33713579

[22] Elkins MR, Robinson M, Rose BR, Harbour C, Moriarty CP, Marks GB, Belousova EG, Xuan W, Bye PT (2006). National Hypertonic Saline in cystic fibrosis (NHSCF) study group. A controlled trial of long-term inhaled hypertonic saline in patients with cystic fibrosis. N Engl J Med.

[23] Ratjen F, Davis SD, Stanojevic S, Kronmal RA, Hinckley Stukovsky KD, Jorgensen N, Rosenfeld M, SHIP Study Group (2019). Inhaled hypertonic saline in preschool children with cystic fibrosis (SHIP): a multicentre, randomised, double-blind, placebo-controlled trial. Lancet Respir Med.

[24] Stahl M, Wielpütz MO, Ricklefs I, Dopfer C, Barth S, Schlegtendal A, Graeber SY, Sommerburg O, Diekmann G, Hüsing J, Koerner-Rettberg C, Nährlich L, Dittrich AM, Kopp MV, Mall MA (2019). Preventive inhalation of hypertonic saline in infants with cystic fibrosis (PRESIS). A randomized, double-blind, controlled study. Am J Respir Crit Care Med.

[25] Robinson M, Regnis JA, Bailey DL, King M, Bautovich GJ, Bye PT (1996). Effect of hypertonic saline, amiloride, and cough on mucociliary clearance in patients with cystic fibrosis. Am J Respir Crit Care Med.

[26] Donaldson SH, Danielle Samulski T, LaFave C, Zeman K, Wu J, Trimble A, Ceppe A, Bennett WD, Davis SD (2020). A four week trial of hypertonic saline in children with mild cystic fibrosis lung disease: effect on mucociliary clearance and clinical outcomes. J Cyst Fibros.

[27] Ferreira ACM, Marson FAL, Cohen MA, Bertuzzo CS, Levy CE, Ribeiro AF, Ribeiro JD (2017). Hypertonic saline as a useful tool for sputum induction and pathogen detection in cystic fibrosis. Lung..

[28] Rosenfeld M, Ratjen F, Brumback L, Daniel S, Rowbotham R, McNamara S, Johnson R, Kronmal R, Davis SD, ISIS Study Group (2012). Inhaled hypertonic saline in infants and children younger than 6 years with cystic fibrosis: the ISIS randomized controlled trial. JAMA..

[29] Dentice RL, Elkins MR, Middleton PG, Bishop JR, Wark PA, Dorahy DJ, Harmer CJ, Hu H, Bye PT (2016). A randomised trial of hypertonic saline during hospitalisation for exacerbation of cystic fibrosis. Thorax..

[30] Buonpensiero P, De Gregorio F, Sepe A, Di Pasqua A, Ferri P, Siano M, Terlizzi V, Raia V (2010). Hyaluronic acid improves "pleasantness" and tolerability of nebulized hypertonic saline in a cohort of patients with cystic fibrosis. Adv Ther.

[31] Brivio A, Conese M, Gambazza S, Biffi A, Tirelli AS, Russo M, Foà M, Colombo C (2016). Pilot randomized controlled trial evaluating the effect of hypertonic saline with and without hyaluronic acid in reducing inflammation in cystic fibrosis. J Aerosol Med Pulm Drug Deliv.

[32] Yang C, Montgomery M (2021). Dornase alfa for cystic fibrosis. Cochrane Database Syst Rev.

[33] Yang C, Montgomery M (2018). Dornase alfa for cystic fibrosis. Cochrane Database Syst Rev.

[34] Southern KW, Clancy JP, Ranganathan S (2019). Aerosolized agents for airway clearance in cystic fibrosis. Pediatr Pulmonol.

[35] Suri R, Metcalfe C, Lees B, Grieve R, Flather M, Normand C, Thompson S, Bush A, Wallis C (2001). Comparison of hypertonic saline and alternate-day or daily recombinant human deoxyribonuclease in children with cystic fibrosis: a randomised trial. Lancet..

[36] Ballmann M, von der Hardt H (2002). Hypertonic saline and recombinant human DNase: a randomised cross-over pilot study in patients with cystic fibrosis. J Cyst Fibros.

[37] Adde FV, Borges KTL, Hatanaka ACF, Nakaie CMA, Cardieri JMA, Oliveira RC (2004). Hypertonic saline X recombinant human DNase: a randomised crossover study in 18 cystic fibrosis patients [abstract]. J Cyst Fibros.

[38] Castellani C, Duff AJA, Bell SC, Heijerman HGM, Munck A, Ratjen F, Sermet-Gaudelus I, Southern KW, Barben J, Flume PA, Hodková P, Kashirskaya N, Kirszenbaum MN, Madge S, Oxley H, Plant B, Schwarzenberg SJ, Smyth AR, Taccetti G, Wagner TOF, Wolfe SP, Drevinek P (2018). ECFS best practice guidelines: the 2018 revision. J Cyst Fibros.

[39] Mogayzel PJ, Naureckas ET, Robinson KA, Mueller G, Hadjiliadis D, Hoag JB, Lubsch L, Hazle L, Sabadosa K (2013). Marshall B; pulmonary clinical practice guidelines committee. Cystic fibrosis pulmonary guidelines. Chronic medications for maintenance of lung health. Am J Respir Crit Care Med.

[40] Lahiri T, Hempstead SE, Brady C, Cannon CL, Clark K, Condren ME, Guill MF, Guillerman RP, Leone CG, Maguiness K, Monchil L, Powers SW, Rosenfeld M, Schwarzenberg SJ, Tompkins CL, Zemanick ET, Davis SD (2016). Clinical practice guidelines from the Cystic Fibrosis Foundation for preschoolers with cystic fibrosis. Pediatrics..

[41] Robinson M, Daviskas E, Eberl S, Baker J, Chan HK, Anderson SD, Bye PT (1999). The effect of inhaled mannitol on bronchial mucus clearance in cystic fibrosis patients: a pilot study. Eur Respir J.

[42] Elkins M, Dentice R (2020). Timing of hypertonic saline inhalation for cystic fibrosis. Cochrane Database Syst Rev.

[43] Dentice R, Elkins M (2021). Timing of dornase alfa inhalation for cystic fibrosis. Cochrane Database Syst Rev.

[44] Hurt K, Bilton D (2014). Inhaled interventions in cystic fibrosis: mucoactive and antibiotic therapies. Respiration..

